# Rattle Snake Oil

**Published:** 1888-05

**Authors:** 


### RATTLE SNAKE OIL.

Rattlesnakes are among the few things that seem to thrive among the rocky hills of Pike county, Pa., and they are just about as plentiful there now as they were when the country was opened. Recently they have become an article of merchandise, owing to the efforts of Anton Hinderman, a little middle-aged German, who leaves his wife and family in Elizabeth, N. J., every year and goes up it Pike county to live in a hut and hunt rattlesnakes. The rattlesnake industry is almost monopolized by Anton. Others occasionally kill a rattler and lie about its length, but the little German hunts for them persistently and methodically, and catches or kills five or ten on every fine day in summer. He sells them alive to showmen and guests at the Pike county hotels occasionally, but his chief income is derived from rattlesnake oil, which he tries out and sells for one or two dollars an ounce, according to the fluctuations of the market.

He catches the rattlers basking on the rocky ledges, and after pinning them down with a forked stick ties strings around their necks and binds them securely in the crotches of the sticks and carries them to his hut, where he puts them in a perforated packing case to await death or sale. He has never been bitten, but he professes to have a botanic cure for snake bites, and says he is not afraid of the biggest rattlesnake in the State.

He does not use fire in extracting the oil, because he believes that it will spoil it. He says the snakes must be hung in the sun and allowed to dry out slowly in its fierce rays, while the oil drips from their tails into wide-mouthed bottles which are suspended to them. A large snake yields several ounces of oil, and it is a very small snake that will not fill an ounce vial with the greenish oil which is reputed to be a sovereign cure for rheumatism and kindred complaints. Country people have great faith in it and country druggists buy all that Anton can furnish. He says that he never puts any other oil in it but he thinks that some of the druggists adulterate it.

Anton was in New York last spring, where he saw the tanned skin of a Florida rattler, nine feet in length. He at once made up his mind to spend next winter where such big rattlers grow, and he thinks he will be able to supply the world with snake oil, snake skins, and live rattlers. He was born in Germany, and educated as a gamekeeper and forester. After he came to this country he was employed at Tuxedo Park. From there he went to Pike county and acted as guide for anglers and hunters who stopped at the various hotels. He learned that there was money in snakes, and having a natural liking for risky sport he became a rattlesnake hunter.—*New York Sun.*

